# Up-regulated MicroRNA-181a induces carcinogenesis in Hepatitis B virus-related hepatocellular carcinoma by targeting E2F5

**DOI:** 10.1186/1471-2407-14-97

**Published:** 2014-02-17

**Authors:** Chengcheng Zou, Yongguo Li, Yiyi Cao, Jinnan Zhang, Jingrong Jiang, Yanrui Sheng, Sen Wang, Ailong Huang, Hua Tang

**Affiliations:** 1 Second Affiliated Hospital, Chongqing Medical University, Chongqing 400016, China; 2 Key Laboratory of Molecular Biology on Infectious Diseases, Ministry of Education, Chongqing Medical University, Chongqing 400016, China; 3 Department of Forensic Medicine, Chongqing Medical University, Chongqing 400016, China; 4 Infection Department of the First Affiliated Hospital, Chengdu University of Traditional Chinese Medicine, Chengdu 610041, China

**Keywords:** HCC, HBV, miR-181a, E2F5, Cell proliferation

## Abstract

**Background:**

Accumulating evidence showed that microRNAs are involved in development and progression of multiple tumors. Recent studies have found that miR-181a were dysregulated in several types of cancers, however, the function of miR-181a in hepatocellular carcinoma (HCC) remains unclear. In this study we assessed the potential association between miR-181a, HBV and HCC.

**Methods:**

The expression of miR-181a in HBV-expressing cells was determined by using qRT-PCR. Dual-Luciferase reporter Assay, qRT-PCR and western blot were performed to investigate the target genes of miR-181a. The effects of miR-181a on HCC proliferation were analyzed by MTS and colony formation assay. Tumor growth assay was used to analyze the effect of miR-181a on tumor formation.

**Results:**

HBV up-regulated miR-181a expression by enhancing its promoter activity. Overexpression of miR-181a in hepatoma cells promoted cell growth *in vitro* and tumor formation *in vivo*. Conversely, inhibition of miR-181a suppressed the proliferation of HBV-expressing cells. Mechanism investigation revealed that miR-181a inhibited the expression of transcription factor E2F5 by specifically targeting its mRNA 3′UTR. Moreover, E2F5 inhibition induced cell growth and rescued the suppressive effect of miR-181a inhibitor on the proliferation of SMMC-7721 cells. Interestingly, we also discovered that HBV could down-regulate E2F5 expression.

**Conclusions:**

Those results strongly suggested that HBV down-regulated E2F5 expression, in part, by up-regulating the expression of miR-181a. Up-regulation of miR-181a by HBV in hepatoma cells may contribute to the progression of HCC possibly by targeting E2F5, suggesting miR-181a plays important role in HCC development.

## Background

Hepatocellular carcinoma (HCC) is the third leading cause of cancer-related deaths around the world. There are about 21,000 new cases diagnosed each year and 700,000 people died due to HCC annually [1,2]. Chronic hepatitis B virus (HBV) infection is the most prominent cause for HCC, which accounts for 55% of cases worldwide and 80% or more of those in the eastern Pacific region and sub-Saharan Africa [3-5]. However, the mechanism by which HBV contributes to the development of HCC remains unclear. Thus, a better understanding of the molecular mechanisms underlying HBV-related HCC will be critical for the improvement of therapeutic strategies for HCC patients.

MicroRNAs (miRNAs) are a novel class of small non-coding RNAs, which play important roles in many of the major biological processes in eukaryotic cells by regulating their target genes post-transcriptionally [6]. Their target genes include numerous regulators of biological processes, such as cell differentiation, proliferation, apoptosis, metabolism, development, and immunity [7,8]. Therefore, miRNAs are implicated in multiple biological processes and aberrant miRNA expression contributes to tumorigenesis and cancer progression [9].

MicroRNA-181a (miR-181a) is a multifunction miRNA that participates in many biological processes such as apoptosis, cell proliferation and cellular invasion [10,11]. In recent years, the miR-181 family was found dysregulated in a variety of human cancers and significantly associated with clinical outcome of cancerous patients [8]. It has been reported that miR-181a significantly overexpressed in a wide variety of cancers, such as gastric cancer [11] and oral squamous cell carcinoma [12]. However, the expression and role of miR-181a in HCC has not yet been characterized. A study of miRNA microarray by our laboratory has shown that miR-181a was significantly increased in HBV-expressing HepG2.2.15 cells compared with HepG2 [13]. This study attempted to evaluate the mechanism of the increasing of miR-181a in HepG2.2.15 as well as the influence of miR-181a overexpression on HCC.

In the present study, we investigated the potential association between miR-181a, HBV and HCC. We examined the expression levels of miR-181a in HCC cell lines and investigate its effect on cell growth. In addition, we also examined the potential role of miR-181a on HCC tumorigenesis in a murine model. Finally, we explored the underlying mechanism of miR-181a functions in HCC. Our study will provide a new perspective in understanding the mechanism of HBV contributing to HCC development.

## Methods

### Cell lines and transfection

The HCC cell lines, HepG2 and SMMC-7721 (Both cell lines are HBV negative) were obtained from the American Type Culture Collection and HepG2.2.15 (HBV positive) was purchased from the Shanghai Second Military Medical University. HepG2 and HepG2.2.15 cell lines were cultured in MEM (Hyclone, China) with 10% FBS (Gibco, USA), 100U/ml penicillin and streptomycin, 5 mmol/L glutamine. SMMC-7721 was cultured in RPMI-1640 (Hyclone, China) supplemented with 100U/ml penicillin and streptomycin. Cells were maintained in a humidified 37°C incubator with an atmosphere of 5% CO2. Transfections were performed with a Lipofectamine 2000 kit (Invitrogen, Carlsbad, CA) according to the manufacturer’s instructions. Transfected cells were harvested at 48 hours.

### Construction of vectors

The miR-181a promoter construct pGL3-miR-181a-P was generated from HepG2 cell genomic DNA. The primers used to amplify the sequence (-800 ~ +240) were 5′-ACGGTACCTGCAGGATCTCAGCAAAGGA-3′ (forward) and 5′- ACCTCGAGAGGAACAGTGAGCAGTAGGA-3′ (reverse). pTARGET-miR-181a vector, which can stable express of miR-181a, contains pri-miR-181a and some of its flanking sequences, and the sequences were amplified by the following primers: 5′-CGCCTCGAGCCCAATATATGTTAATCTCTTACC-3′ (forward) and 5′-GCGCGCGTCGACTTTTTAATAAATTTTTACTTGCTA-3′ (reverse). The 3′untranslated regions (3′-UTRs) of E2F5 containing an intact miR-181a recognition sequence were amplified by PCR from genomic DNA, and the PCR product was subcloned into a pGL3-control vector (Promega, Madison, WI) immediately downstream of the luciferase gene. The primers used were 5′-ATTCTAGATGGGACTGTTATCTACCT-3′ (forward) and 5′-ACTCTAGAGATCCTCGTTTACATCCTTCA-3′ (reverse). A pGL3 construct containing the E2F5 3′-UTR with point mutations in the seed sequence was amplified from the wild vector. The primers used were 5′-CATATGATTCTGTAGTAGACCGACAATCAGTGTATG-3′ (forward) and 5′-CATACACTGATTGTCGGTCTACTACAGAATCATATG-3′ (reverse). MiR-181a inhibitor, siE2F5 and their negative controls (NC) were purchased from Invitrogen. Sequences were as follows: miR-181a inhibitor: ACUCACCGACAGCGUUGAAUGUU. siE2F5: sense: 5′-GAGGUACCCAUUCCAGAAATT-3′, antisense: 5′-UUUCUGGAAUGGGUACCUCTT-3′. Ad-HBV adenovirus and its control Ad-GFP adenovirus were constructed by our laboratory as following steps: HBV 1.3 fold genome was ligated into shuttle vector pAdTrack-TO4, then pAdTrack-TO4-HBV1.3 was linearized by *Pme*I and transfected into BJ5183 cells containing pAd-Easy1 to form a recombinant plasmid. After that the confirmed recombinant plasmid was linearized by *Pac*I restriction endonuclease and transfected into HEK-293 cells to generate recombinant adenoviruses [14].

### Stable cell generation

SMMC-7721 cells were transfected with pTARGET-miR-181a or pTARGET and selected with G418 (1000ug/ml). Stable cell line p-miR-181a, which could stable express miR-181a, was established.

### Cell proliferation assay

SMMC-7721 cells were trypsinized and seeded into 96-well culture plates 24 h after transfection with a density of 7000 cells/well. The cells were harvested at different time points (24, 48, 72, and 96 h) for growth assay using the MTS kit (cellTiter96AQ, Promega, USA) following the manufacturer’s protocol and the absorption was read at 490 nm.

### Colony formation assay

Twenty-four hours after transfection, cells were trypsinized and seeded into 6-well plates with a density of 2000 per well. When the cells grew to visible colonies, the colonies were washed once with PBS and fixed with 4% paraformaldehyde for 20 min. Next, fixed colonies were washed again with PBS, and stained with crystal violet for 20 min. Finally, the crystal violet dye was washed with PBS. The number of colonies was counted under a microscope.

### Luciferase reporter assay

For the luciferase reporter assay, cells were cotransfected with 300 ng of pGL3-E2F5-WT or pGL3-E2F5-Mut constructs and 500 ng pTARGET-miR-181a or negative control. Each sample was cotransfected with 50 ng of pRL-TK plasmid expressing renilla luciferase (Promega, Madison, WI). Cells were collected 48 h after transfection and analyzed using the Dual-Luciferase Reporter Assay System (Promega, Madison, WI). Relative luciferase activity was normalized to renilla luciferase activity. Transfections were done in duplicate and repeated at least 3 times in independent experiments.

### Reverse-transcription reaction and quantitative real-time PCR

Total RNAs were extracted with TRIzol reagent (Invitrogen, Carlsbad, CA). To analyze miR-181a expression, miRNA cDNA Kit (CWBIO, China) and miRNA Real-Time PCR Assay Kit (CWBIO, China) were used. And the miRNA specific forward primer was 5′-AACATTCAACGCTGTCGGTGAGT-3′ and a universal reverse primer was provided by miRNA Real-Time PCR Assay kit. U6 was used as a miRNA internal control, the primers for U6 were 5′-AGAGCCTGTGGTGTCCG-3′ (forward) and 5′-CATCTTCAAAGCACTTCCCT-3′ (reverse). To measure messenger RNA (mRNA) level of E2F5, total RNA was reversely transcribed using the Reverse Transcription System (Promega, Madison, WI). The primers used were 5′-TCAGGCACCTTCTGGTACACAACT-3′(forward) and 5′- AGCAGCACATGGATAGGTCCTGAA-3′(reverse). β-actin was used as an endogenous control, the primers for it were 5′-GTGGATCAGCAAGCAGGAGT-3′ (forward), 5′-TGTGTGGACTTGGGAGAGGA-3′ (reverse). All quantitative real-time polymerase chain reaction (qRT-PCR) samples were performed by using SYBR Green PCR master mix (CWBIO, China). The real-time PCR reactions were performed in triplicate and included no-template controls. Relative changes in gene expression were calculated using the 2^-ΔΔCT^ method [15].

### Western blot analysis

The cells were lysed with 1% RIPA Lysis Buffer (Beyotime, China) 48 h after transfection. The supernatants were collected, and protein concentration was determined using the BCA Assay Kit (Beyotime, China). The protein samples were separated analyzed by 10% SDS-PAGE and then transferred to a PVDF membrane. The membrane was blocked with 5% milk, followed by an overnight incubation at 4°C with a primary rabbit antibody against human E2F5 (Bioword, USA). The membrane was washed three times in TBST and then incubated with a goat anti-rabbit HRP secondary antibody. Last, the bound antibody was detected by chemiluminescence with the ECL Detection Reagent (Millipore, Billerica, MA). The data were normalized to β-actin.

### Tumor growth assay

Female BALB/c nude mice (4-6 weeks old) were purchased from the Laboratory Animal Services Center of CUHK. Animal handling and experimental procedures were approved by the Animal Experimental Ethics Committee of CUHK. A total of 5 × 10^6^ p-miR-181a cells, were injected subcutaneously (SC) into the dorsal flank of nude mice. Each group contained 5 mice. Tumor size was measured every 2 to 3 days. 2 weeks later, mice were sacrificed and tumors were dissected.

### Immunohistochemistry

Paraformaldehyde-fixed, paraffin-embedded tissues of transplanted tumors were sectioned at 4.5 μm thickness and analyzed for Ki-67 (Bioword, 1:50 dilution) and E2F5 (Bioword, 1:100 dilution) expression. Visualization was achieved by using the 3,3′-diaminobenzidine substrate. Sections stained with PBS only were used as the negative staining control.

### Statistical analysis

Data are expressed as mean standard deviation (SD). Statistical analysis was performed by using the independent *t*-test. *P* value of less than 0.05 was considered statistically significant.

## Results

### HBV up-regulated the expression of miR-181a by enhancing its promoter activity

**Figure 1:**
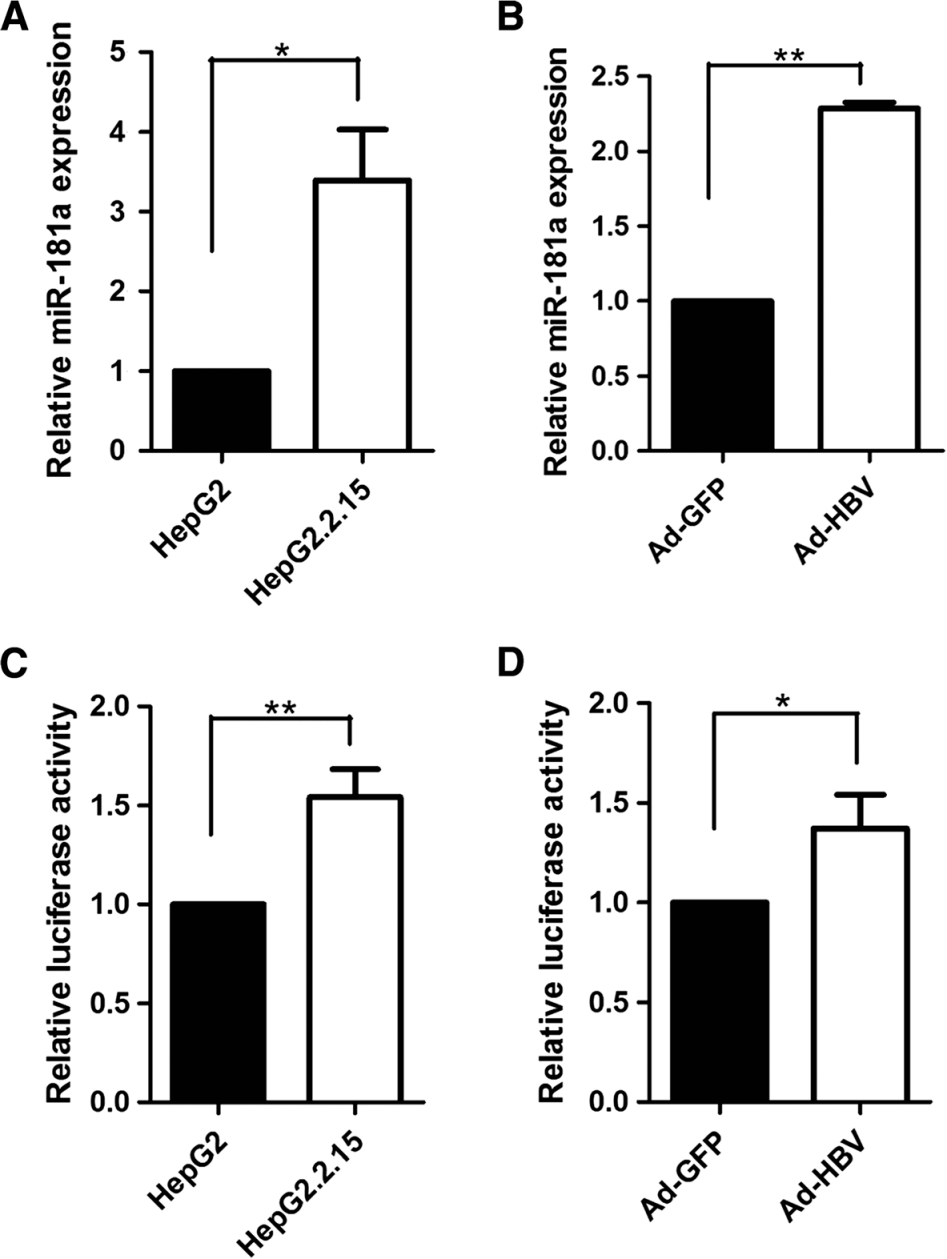
**HBV up-regulated the expression of miR-181a by increasing its promoter activity. (A)** Relative expression of miR-181a in HepG2 and HepG2.2.15. **(B)** Relative expression of miR-181a in Ad-HBV or Ad-GFP infected HepG2 cells. miRNA abundance was normalized to U6 RNA. **(C)** Dual luciferase reporter analysis was performed to analyze miR-181a promoter activity in HepG2.2.15 and HepG2 cells. **(D)** miR-181a promoter activity in HepG2 cells with Ad-GFP or Ad-HBV infection. *P < 0.05, **P<0.01.

Firstly, the expressions of miR-181a in a panel of cell lines including HepG2, HepG2.2.15 (a HBV stably transfected cell lines constitutively producing HBV) and HepG2 cells infected with recombinant adenoviruses expressing HBV (Ad-HBV) or GFP (Ad-GFP) were analyzed, and we found that miR-181a expressions were markedly higher in the HBV-exprssing cells (HepG2.2.15 and Ad-HBV) when compared to control cells (Figure 1A, B). These results were consistent with our previous miRNA microarray data and illustrated that HBV could up-regulate miR-181a expression. To further investigate the mechanism of up-regulation miR-181a by HBV, the activity of miR-181a promoter was compared in HepG2.2.15 and HepG2 cells by using luciferase reporter assay. The results showed that HBV replication enhanced the promoter activity of miR-181a (*P* = 0.0084) (Figure 1C). Consistently, the promoter activity of miR-181a significantly higher Ad-HBV-HepG2 cells compared with Ad-GFP cells (*P* = 0.0266) (Figure 1D). These results suggested that HBV could up-regulate the expression of miR-181a by increasing its promoter activity.

### MiR-181a overexpression induced cell proliferation in HCC cells

**Figure 2:**
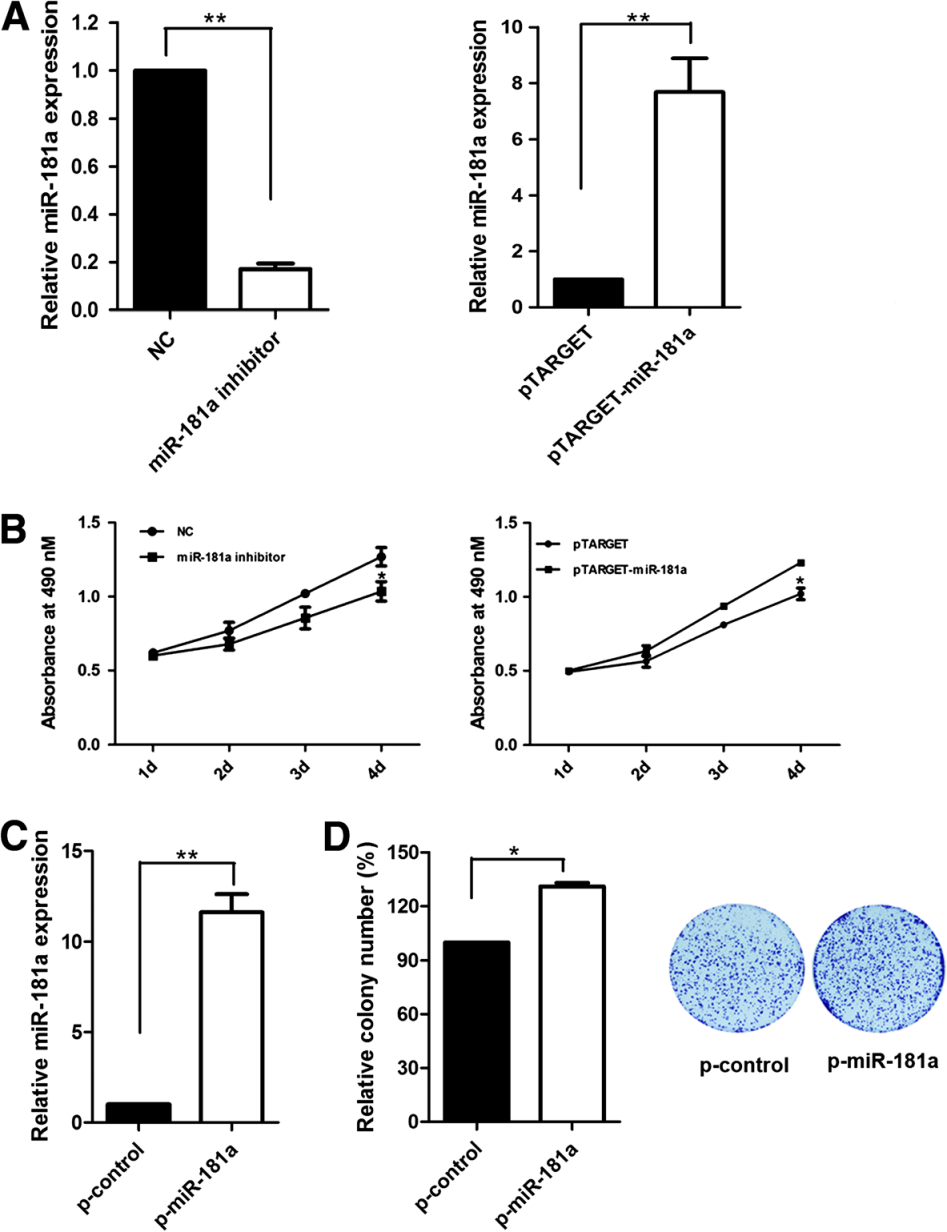
**MiR-181a overexpression induced cell proliferation in HCC cells. (A)** SMMC-7721 cells were transfected with pTARGET-miR-181a, miR-181a inhibitor and their negative controls. 48 h after transfection, expression of miR-181a was measured by qRT-PCR. **(B)** Effect of miR-181a on cell proliferation was measured by MTS assay in pTARGET-miR-181a or miR-181a inhibitor transfected SMMC-7721 cells. **(C)** Relative miR-181a expression in p-miR-181a which can stably express miR-181a. miRNA abundance was normalized to U6 RNA. **(D)** Representative pictures of colony formation assay of p-miR-181a and p-control cells. Colonies were evaluated and values were reported as the ratio. *P < 0.05, **P<0.01.

To determine whether miR-181a could affect cell proliferation, SMMC-7721 cells were transiently transfected with pTARGET-miR-181a, miR-181a inhibitor or their negative controls and then cell growth was measured. Compared with the NC control, the miR-181a level was decreased nearly 6 times in SMMC-7721 cells transfected with miR-181a inhibitor (*P* = 0.0002) (Figure 2A). On the other hand, transfection with pTARGET-miR-181a increased miR-181a expression approximately 8 times, compared with control (*P* = 0.0068) (Figure 2A). The inhibition of miR-181a resulted in a decrease in cell number over a course of 4 days (*P* = 0.0108). In contrast, the overexpression of miR-181a significantly promoted cell proliferation (*P* = 0.0124) (Figure 2B). Furthermore, Clone formation assay was performed in cells stably express miR-181a (Figure 2C). Overexpression of miR-181a increased the number of colonies, compared with control (*P* = 0.0357) (Figure 2D).

### E2F5 was a target gene of miR-181a

**Figure 3:**
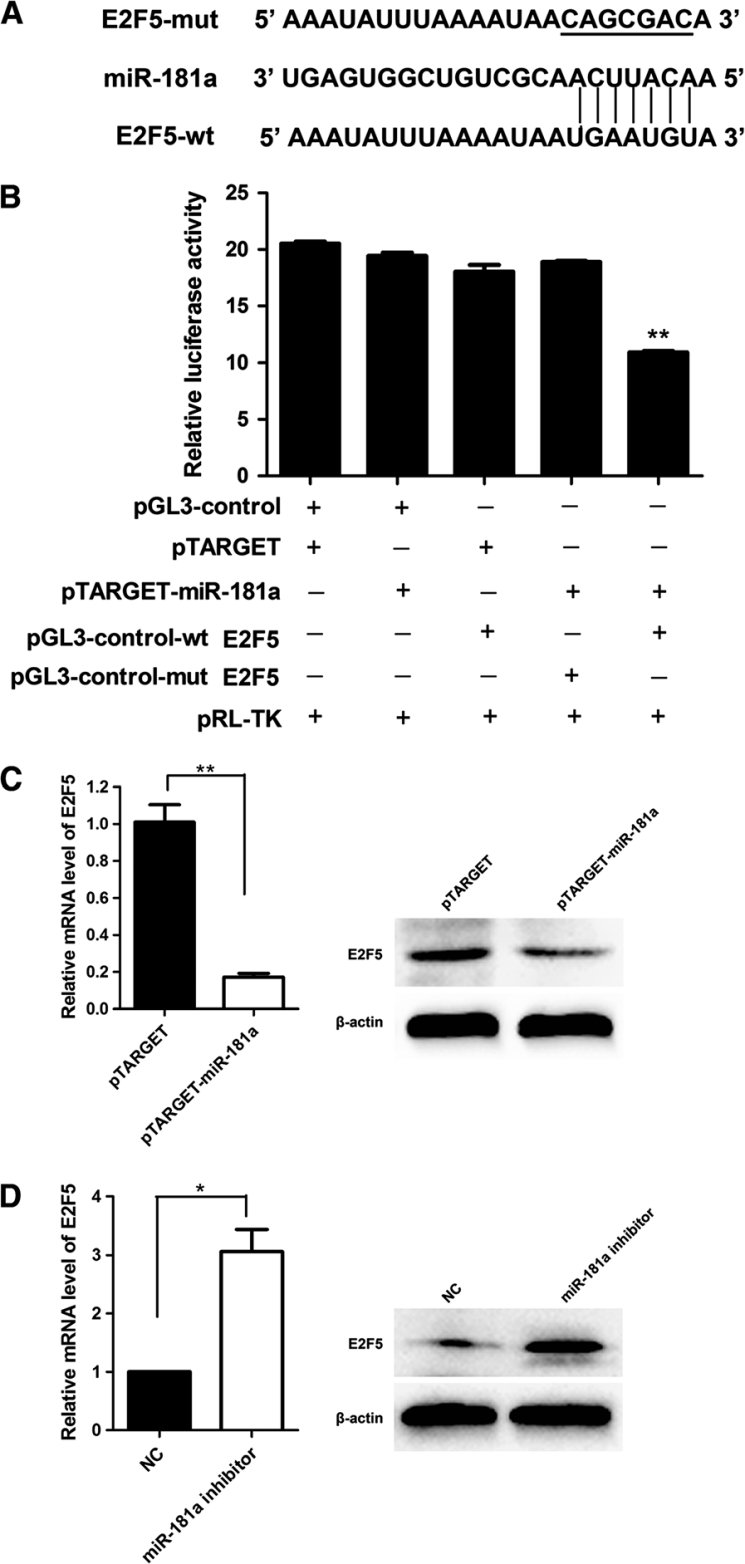
**E2F5 was a target gene of miR-181a. (A)** Schematic diagram of predicted miR-181a binding site in the E2F5 3′-UTR. **(B)** Luciferase reporter assays in SMMC-7721 cells, with cotransfection of wt or mut E2F5 3′-UTR and miRNA as indicated. **(C)** E2F5 mRNA and protein expression levels in SMMC-7721 cells transfected with pTARGET-miR-181a or its respective control. **(D)** E2F5 mRNA and protein expression levels in SMMC-7721 cells transfected with miR-181a inhibitor or its negative control. β-actin was used as an internal quantitative control. *P < 0.05, **P<0.01.

To explore the possible mechanisms of growth regulation by miR-181a, miR-181a targets were analyzed by using the TargetScan and MiRanda bioinformatics algorithm. Software analysis revealed that E2F5 might be a potential target of miR-181a based on putative target sequences of the E2F5 3′-UTR (Figure 3A). We further performed luciferase assay in SMMC-7721 cells to determine whether miR-181a could directly target 3′UTR of E2F5 in cells. The target sequence of E2F5 3′UTR (E2F5-WT) or mutant sequence (E2F5-Mut) was cloned into luciferase vector. The results showed that miR-181a significantly decreased the luciferase activity of the E2F5 3′-UTR but not mutant sequence in SMMC-7721 cells (*P* = 0.0014) (Figure 3B).Moreover, overexpression of miR-181a significantly down-regulated E2F5 expression at both the mRNA and protein levels in SMMC-7721 cells (*P* = 0.0009) (Figure 3C). In contrast, inhibition of miR-181a resulted in an enhancement of E25F5 mRNA and protein levels (Figure 3D). Taken together, these data strongly suggest that E2F5 was a direct target of miR-181a.

### E2F5 was involved in miR-181a inducing promotion of HCC cells proliferation

**Figure 4:**
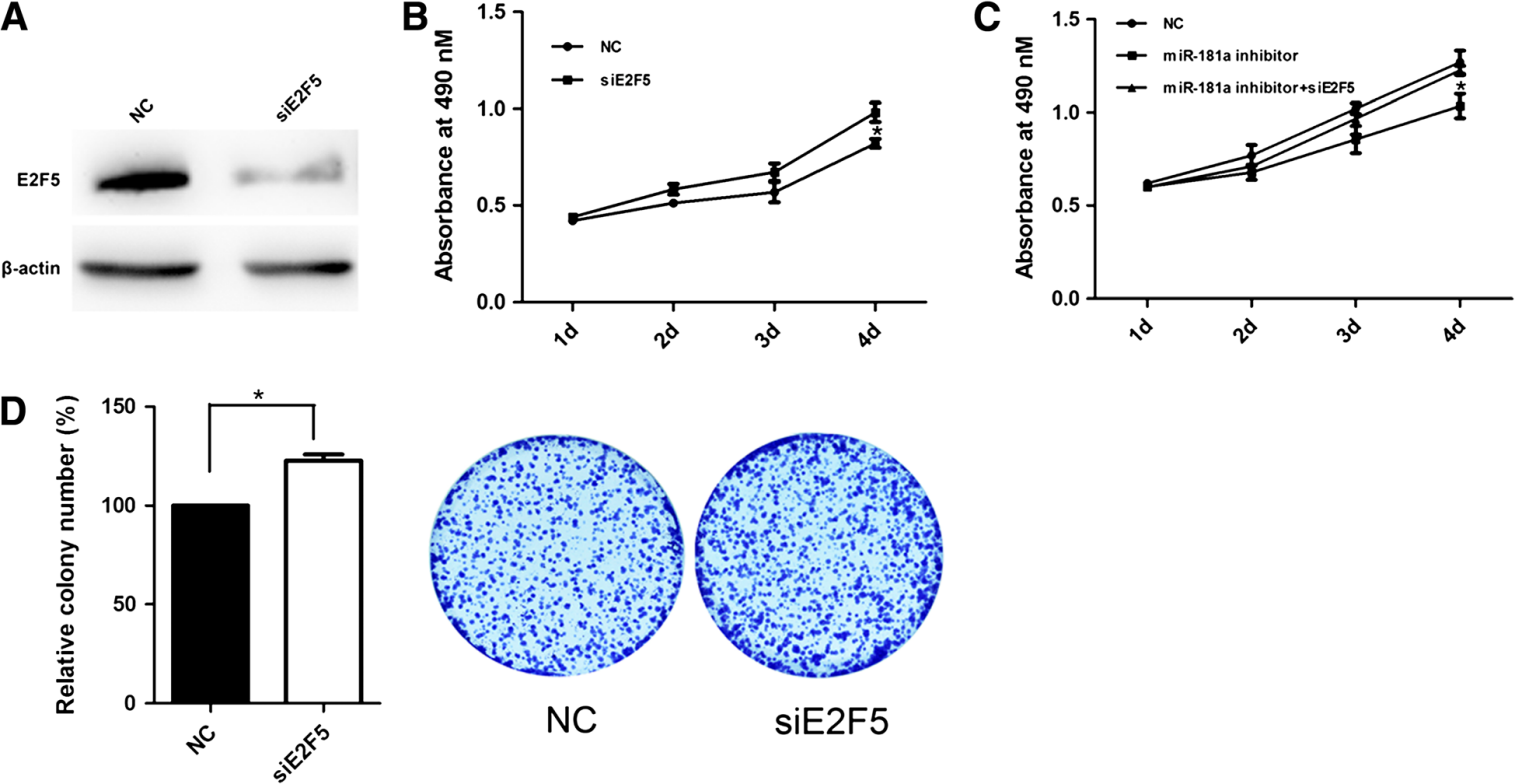
**E2F5 was involved in miR-181a inducing promotion of HCC cells proliferation. (A)** Western blot was performed to analyze E2F5 expression in siE2F5 transfected SMMC-7721 cells. β-actin was used as an internal quantitative control. **(B)** Effect of siE2F5 on cell proliferation was measured by MTS assay. **(C)** SMMC-7721 cells were transfected with NC, miR-181a inhibitor or miR-181a inhibitor and siE2F5, and then MTS assay was performed. **(D)** Representative pictures of colony formation assay of siE2F5 transfected cells. Colonies were evaluated and values were reported as the ratio between siE2F5 transfected cells and NC transfected cells. *P < 0.05.

To elucidate whether the growth-promoting effect of miR-181a was mediated by repression of E2F5 in HCC cells, the effect of E2F5 on cell growth was examined. First, SMMC-7721 cells were transfected with siRNA against E2F5 or the negative control and then we examined cell growth by MTS assay and colony formation assay (Figure 4A). MTS assay results showed that gene silencing of E2F5 promoted cell proliferation of SMMC-7721 cells (Figure 4A, B). Moreover, the inhibitory effect of miR-181a inhibitor on cell growth was reversed when E2F5 expression was knockdown (Figure 4C). Colony formation assay showed that E2F5 silencing significantly increased the number of colonies (Figure 4D), similar to those induced by miR-181a. Taken together, these findings indicated that E2F5 was a functionally important target of miR-181a which was involved in the proliferation of HCC cells.

### HBV down-regulated the expression of E2F5

**Figure 5:**
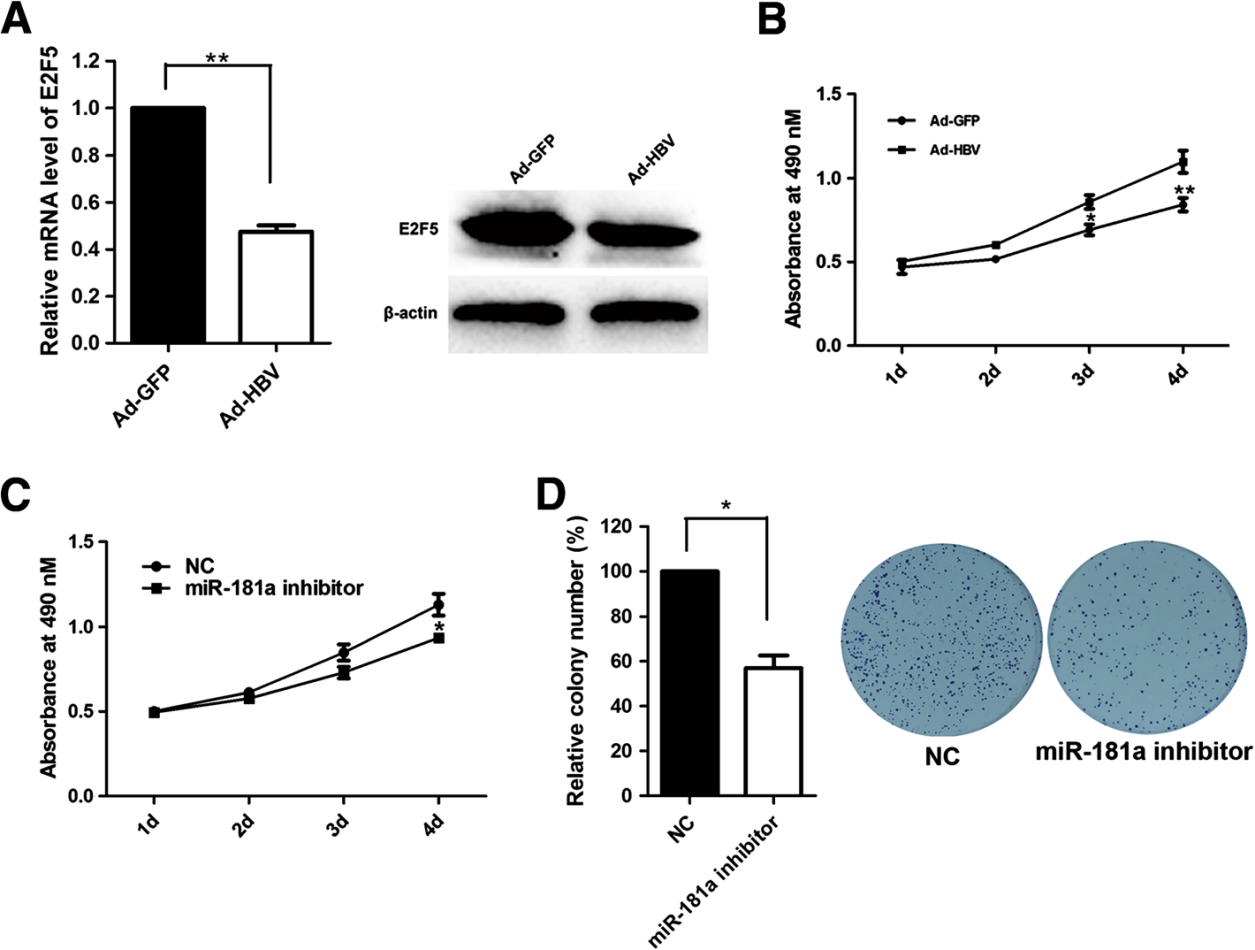
**HBV down-regulated the expression of E2F5. (A)** Relative E2F5 mRNA and protein levels in Ad-GFP or Ad-HBV infected HepG2 cells. **(B)** Effect of HBV on cell proliferation was measured by MTS assay in HepG2 cells infected with Ad-HBV. **(C)** HepG2.2.15 cells were transfected with NC or miR-181a inhibitor, and then MTS assay was performed. **(D)** Representative pictures of colony formation assay of miR-181a inhibitor transfected HepG2.2.15 cells. Colonies were evaluated and values were reported as the ratio between miR-181a inhibitor transfected cells and NC transfected cells. **P<0.01.

The above results prompted us to examine whether HBV could regulate the expression of E2F5. Therefore, the expression levels of E2F5 were measured in HepG2 cells infected by Ad-HBV or Ad-GFP. E2F5 was down-regulated in Ad-HBV infected cells in comparison with the Ad-GFP control group (*P* = 0.0049) (Figure 5A). The result demonstrated that HBV was inversely correlated with E2F5 level.

It has been reported that the growth-promoting effect of HBV plays an important role in the progression of HBV-related HCC [16,17]. In this study, HBV was proved to promote cell growth of hepatoma by MTS (*P* = 0.0045) (Figure 5B). To investigate whether HBV promoted cell growth through regulating miR-181a expression, miR-181a inhibitor was transfected into HepG2.2.15 cells and then cell growth was analyzed. The results revealed that miR-181a inhibited HepG2.2.15 cells resulted in a decrease of cell viability (*P* = 0.0169) (Figure 5C). Furthermore, inhibition of miR-181a also reduced the numbers and size of HepG2.2.15 cells colonies as determined by colony formation assay (*P* = 0.0289) (Figure 5D). Our studies found a new pathway that HBV promoted cells proliferation, by up-regulating miR-181a expression and down-regulating E2F5 expression.

### MiR-181a promoted tumor growth of SMMC-7721 cells in nude mice

**Figure 6:**
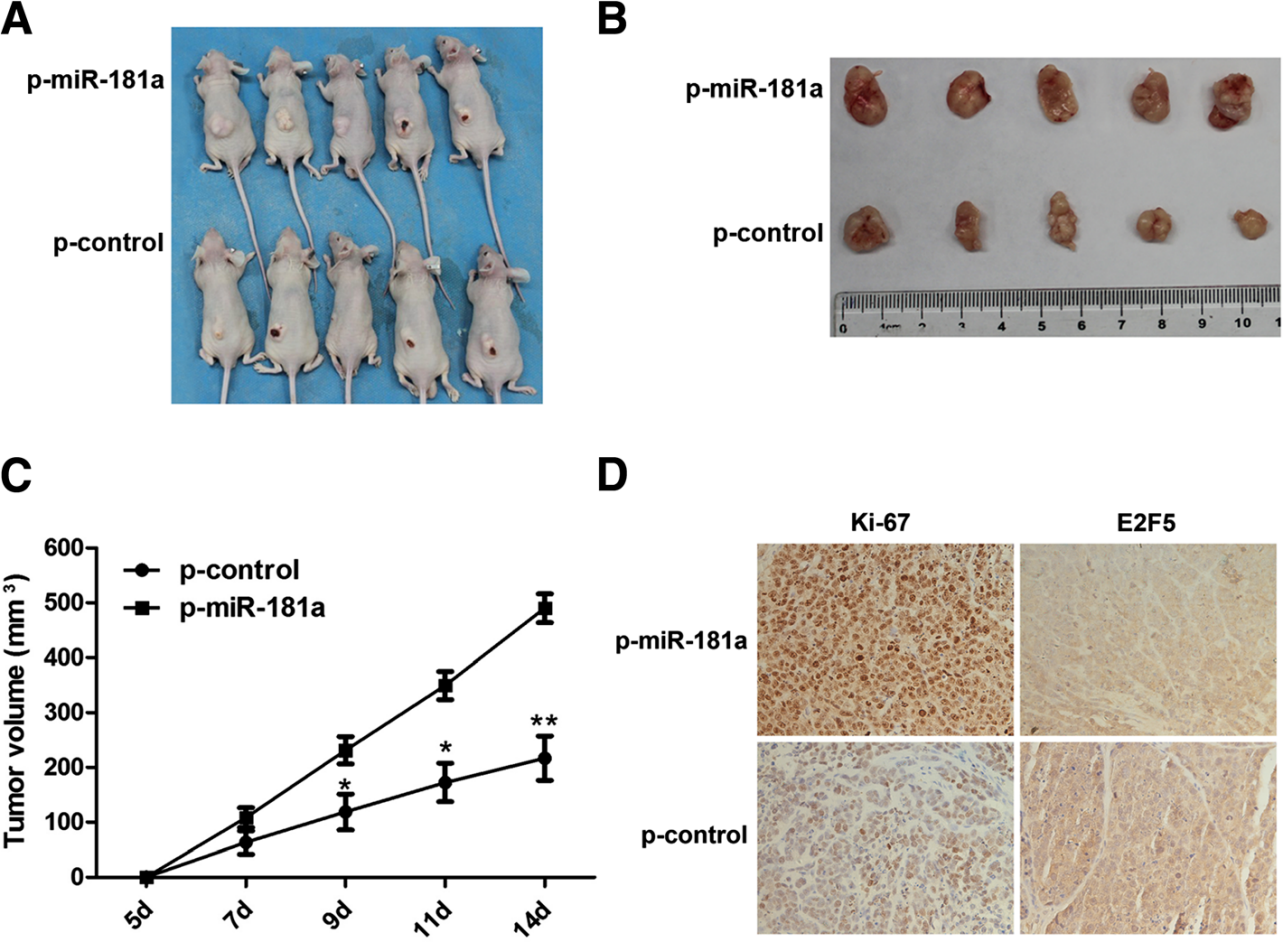
**MiR-181a promoted tumor growth of SMMC-7721 cells in nude mice. (A)** P-miR-181a cells produced larger tumors than control cells at 14 days after implantation. **(B)** Representative picture of tumors formed. **(C)** Growth curve of tumor volumes. Each piece of data represented the mean ± SD of 5 mice. **(D)** Ki-67 and E2F5 stained sections of transplanted tumors. *P < 0.05, **P < 0.01.

P-miR-181a or p-control cells were SC injected into the dorsal flank of nude mice. At 2 weeks after inoculation, those mice injected with p-miR-181a cells carried larger burdens (Figure 6A, B). As early as 9 days postimplantation, the growth of tumor between two groups became statistically significant. Notably, the average tumor volume of the p-miR-181a group was markedly larger than that of control group (*P* = 0.0006) (Figure 6C).

For a better understanding of the molecular mechanism of miR-181a on tumorigenesis *in vivo*, the expression of Ki-67 and E2F5 were examined in tumor tissues by using immunohistochemical analysis. The results showed the staining intensity and the number of Ki-67 positive tumor cells were increased in p-miR-181a treated group when compared with control (Figure 6D). The E2F5 positively stained cells was marked decreased in p-miR-181a treated group, compared with control (Figure 6D). These results strongly suggested that miR-181a promoted tumorigenesis of hepatoma cells *in vivo* by regulating E2F5 expression.

## Discussion

HCC develops through a multistep carcinogenic process, affecting several tumorigenic-related genes by genetic or epigeneticchanges [18] and HBV is widely accepted to be a main cause of HCC. In recent years, miRNAs have been reported frequently to be involved in many biological events, especially tumorigenesis [6,19]. Zhang *et al.* have reported that several miRNAs were up-regulated in HepG2.2.15 cells, including miR-181a [13]. Is there any relationship among HCC, HBV and miR-181a?

Our study firstly showed that up-regulation of miR-181a in HBV-related HCC cell lines and HBV could induce miR-181a expression by enhancing its promoter activity. It is therefore possible that miR-181a might contribute to the carcinogenesis of HBV-related HCC. Various studies have shown that the dysregulated of miR-181a was associated with a variety of human cancers and participates in the occurrence of multiple human cancers. For example, Zhu *et al*. reported that miR-181a improves proliferation and invasion and suppresses apoptosis of osteosarcoma cell [20]. However, whether miR-181a plays a role in the development of HCC has not been addressed. The main topic of this study is the target gene(s) of miR-181a and the influence of the up-regulation miR-181a by HBV on HCC.

Numerous studies show that miR-181a could promote various cancer cell growths, such as gastric cancer [11], osteosarcoma [20]. Can miR-181a promote the growth of hepatoma cells? Our study pointed out that miR-181a could improve hepatoma cell proliferation in *vitro* and tumor growth in *vivo.* These results suggested that miR-181a acted as an oncogene in HCC.

The E2F5 protein, a transcription factor, is a key regulator of the cell growth [21]. We firstly identified E2F5 as a direct target gene of miR-181a. Silence E2F5 could improve cell proliferation and rescued the suppressive effect mediated by miR-181a inhibitor. MiRNA usually affect several target genes, thus other genes besides E2F5 could also be affected by miR-181a and contributing to the increase of cell proliferation. For example, the ATM, TGFBRAP1, and CCNT2 genes could also be the putative targets of miR-181a and worthy to be investigated.

It has been reported that HBV plays an important role in promoting cell growth [16,17]. Our study demonstrated that HBV could promote hepatoma cell proliferation by down-regulating E2F5 expression. MiR-181a inhibitor could suppress the growth of HepG2.2.15 cells. All these findings suggest that HBV promotes cell growth by up-regulating miR-181a expression and down-regulating E2F5 expression. Our study found a novel mechanism for the growth- promoting effect of HBV.

However, further research is still needed to provide a better understanding of the function and mechanism of miR-181a in HCC, such as the effect of miR-181a on invasion, migration, metastasis, apoptosis in HCC cells. Moreover, those results are needed further examined in HCC samples. In this study, we also examined the effect of E2F5 on cell cycle progression, but no difference was observed between siE2F5 transfected cells and its negative control (data not shown). Qin *et al.* have reported that overexpression of E2F5/p130, but not E2F5 alone, can inhibit E2F-induced cell cycle entry [22]. So E2F5 inhibition alone might not effect cell cycle progression. The mechanism of the effect of E2F5 and miR-181a on cell proliferation needs further study. Also, whether HBV enhanced the activity of miR-181a promoter through affecting a certain transcription factor is worthy to investigate.

## Conclusions

The key findings of the current study were that miR-181a could promote cell proliferation in *vitro* and tumor formation in *vivo* by targeting E2F5. HBV could negatively regulate miR-181a and E2F5 expression. The inhibition of miR-181a could suppress the growth-promoting effect of HBV. These data indicated that miR-181a played an essential role in the regulation of HCC cell proliferation and may function as an onco-miRNA in HBV-related HCC.

## Competing interests

The authors declare that they have no competing interests.

## Authors’ contributions

CZ and YL conducted the experiments and prepared the manuscript, under the supervision of HT, who also designed the study. AH gave technical advice, YC, JJ and JZ contributed with the acquisition of data and also provided clinical advice during manuscript preparation. YS and SW revised the final text. All authors read and approved the final manuscript.

## Pre-publication history

The pre-publication history for this paper can be accessed here:

http://www.biomedcentral.com/1471-2407/14/97/prepub
